# Frailty and Sarcopenia Assessment in Patients with Advanced Chronic Liver Disease in a Tertiary Center in Romania

**DOI:** 10.3390/diagnostics15010016

**Published:** 2024-12-25

**Authors:** Petruta Violeta Filip, Denisa Cuciureanu, Corina Silvia Pop, Andreea Nicoleta Marinescu, Florentina Furtunescu, Laura Sorina Diaconu

**Affiliations:** 1 Department of Internal Medicine and Gastroenterology, Carol Davila University of Medicine, 020021 Bucharest, Romaniasorina.diaconu@umfcd.ro (L.S.D.); 2 Departments of Internal Medicine and Gastroenterology, Bucharest University Emergency Hospital, 050098 Bucharest, Romania; florentina.furtunescu@umfcd.ro; 3 Department of Radiology, Bucharest University Emergency Hospital, 050098 Bucharest, Romania; 4 National Institute of Public Health, 050463 Bucharest, Romania

**Keywords:** advance chronic liver disease, cirrhosis, sarcopenia, frailty, outcome

## Abstract

**Background/Objectives**: Sarcopenia and frailty are both multidimensional and interrelated problems for patients with cirrhosis and require prompt assessment and appropriate management because of their impact on disease outcomes. Our purpose is to identify the prevalence of sarcopenia and frailty in patients with advanced liver disease. Furtherksdnvk more, our purpose is to explore the association between sarcopenia, frailty, and various complications and the impact of these conditions on short- and long-term hospital survival rates. **Methods**: A prospective, observational, unicentric study was conducted in an emergency university hospital in Romania between January 2021 and December 2023 that included patients with advanced liver diseases. The patients with sarcopenia and frailty were selected using measurements of handgrip strength (HGS), Short Physical Performance Battery (SPPB), liver frailty index (LFI), and skeletal muscle index (SMI). Patients were divided into four groups based on the presence of sarcopenia and/or frailty. **Results**: This study included 128 patients. Younger patients associated with both sarcopenia and frailty (55.76 ± 10.46 years). Most males were without sarcopenia and frailty (63.93%) compared to those with both sarcopenia and frailty (36.07%). The Child–Pugh score C was identified in the majority of those with both sarcopenia and frailty (69.70%). Higher values for MELD-Na scores were obtained in the group with sarcopenia and frailty (25.45 ± 6.924). Biomarkers like albumin, sodium, C-reactive protein, bilirubin, and platelets were statistically significant as mortality predictors in all four groups. Patients with both sarcopenia and frailty presented more often with encephalopathy and spontaneous bacterial peritonitis. Survival rates in the short and long term were lower for the patients who associated both sarcopenia and frailty compared to those without sarcopenia and frailty. **Conclusions:** The presence of sarcopenia and frailty significantly impacts outcomes in patients with decompensated advanced liver disease. When both conditions coexist in the same patient, they markedly increase in-hospital mortality, as well as short- and long-term survival rates.

## 1. Introduction

Advanced chronic liver disease remains a serious public health problem globally, causing more than 2 million deaths annually, 50% of which are secondary to cirrhosis [1,2,3,4,5,6]. In 2023, in Romania, advanced chronic liver diseases registered a prevalence of 17.9% [4]. Unfortunately, chronic liver diseases affect patient quality of life by reducing their ability to perform daily activities [2,6,7,8,9].

Reversibility of liver fibrosis is not currently possible, and liver transplantation remains the only therapeutic option for patients with end-stage cirrhosis or hepatocellular carcinoma [10,11,12]. However, most patients are not suitable for transplantation due to disease complications [10,11,12].

Prognostic scores such as Child–Pugh and MELD-Na play a significant role in assisting healthcare providers in the management of liver disease [13,14]. However, it is crucial to recognize that we frequently fail to adequately consider an individual patient’s clinical condition when making treatment decisions, which can harm the patient’s overall prognosis [5,6,8,15,16,17,18]. The impairment of physical function represents a complex physiological system that increases a patient’s vulnerability to various stress factors and, consequently, may lead to poor outcomes. Because of this, hepatologists have started to pay more attention to the assessment of frailty and sarcopenia in cirrhotic patients [15,16,18,19,20].

Chronic impairment of liver function leads to complications such as frailty and sarcopenia, conditions that significantly impact individuals’ overall health and quality of life [16,18,20].

Frailty is a dynamic condition characterized by impaired physical function, decreased functional performance, and disability due to decreased muscle contractile function [20,21,22]. The Fried frailty phenotype is defined by the presence of three of five criteria: involuntary weight loss of more than 5% of total body weight in the past year, decreased grip strength (HGS), reduced walking speed, reduced physical activity (questionnaire), and fatigue/exhaustion (questionnaire) [20,21,23].

This term is distinct from malnutrition (nutritional imbalance) and from sarcopenia, which is defined by the loss of muscle mass [20,24]. Sarcopenia is characterized by decreased skeletal muscle mass associated with reduced muscle strength and/or decreased physical performance [5,16,20,23,24,25].

This paper aims to identify the prevalence of sarcopenia and frailty in a select group of patients with cirrhosis who were admitted to an emergency hospital. Additionally, it explores the association between sarcopenia and frailty, aiming to reveal any significant relationships with various complications that may arise from these conditions. Furthermore, this study investigates the impact and relevance of these conditions on survival rates during hospitalization at 30 days, 90 days, 180 days, and 12 months, as well as hospital readmission rates at 30 days.

## 2. Materials and Methods

### 2.1. Study Design and Population Selection

This prospective, observational, unicentric study was conducted in the Department of Internal Medicine 2 of the Bucharest University Emergency Hospital between January 2021 and December 2023. It included 128 patients diagnosed with advanced liver disease.

The local Ethics Committee approved the study protocol, which was conducted according to the 1975 Declaration of Helsinki after each patient gave informed consent to participate (Protocol No 58134/16.11.2020).

The diagnosis of advanced liver disease was based on physical examination, laboratory tests, and imaging tests [abdominal ultrasound, liver elastography, upper digestive endoscopy, and computed tomography (CT)].

Patients were included in this study based on the following inclusion criteria:

(1) over 18 years of age;

(2) informed consent;

(3) diagnosis of advanced liver disease;

(4) CT scan at diagnosis or during hospitalization.

The exclusion criteria were as follows:

(1) the inability to follow instructions or walk without assistance;

(2) the presence of any of the factors that could have independently influenced sarcopenia: chronic infections (tuberculosis, human immunodeficiency virus), inflammatory bowel diseases, neuromuscular diseases, chronic kidney disease, chronic heart failure, COPD, or malignant tumors (except hepatocarcinoma).

Data collected from medical records included age, sex, etiology of chronic liver disease, anthropometric assessment, hemoglobin value, platelets, liver chemistry, C-reactive protein, INR, electrolytes, Child–Pugh score, MELD-Na score, and the presence of portal hypertension (esophageal varices, portal gastropathy, ascites), upper gastrointestinal bleeding, infections (such as spontaneous bacterial infection, urinary infection, and pneumonia), hepatic encephalopathy, hepatorenal syndrome, and hepatocellular carcinoma. We also collected data about the length of the hospital stay (days), the in-hospital mortality, if there was a readmission in the first 30 days after discharge, and the survival from baseline to 30 days, 90 days, 180 days, and 12 months.

Anthropometric measurements included body mass index (BMI) and handgrip strength (HGS), which were measured using a dynamometer on the dominant hand. The test was performed three times with a 30 s pause between each test. The highest recorded value was used. All values were recorded in kilograms. The cut-off thresholds were those from the European Working Group on Sarcopenia in Older People 2 (EWGSOP): less than 27 kg for men and less than 16 kg for women [24].

We also performed the Short Physical Performance Battery (SPPB), which includes balance tests, gait speed tests, and chair stand tests.

We also used the liver frailty index (LFI), which includes HGS measurements, balance tests, and chair stand tests.

All patients in this study performed a computed tomography (CT) scan, and their skeletal muscle index (SMI) at the third lumbar vertebra was measured by a single observer (experienced radiologist).

The diagnosis of frailty was made by the presence of three out of five criteria. The diagnosis of sarcopenia was made by the presence of decreased HGS, decreased physical performance, and the measurement of SMI on the CT scan.

### 2.2. Statistical Analysis

The statistical analysis included both elements of descriptive statistics (frequency, percentage, mean, standard deviation, median) and elements of inferential statistics. The Shapiro–Wilk test was applied to determine the distribution of the analyzed data series. To compare 2 data sets with non-Gaussian distributions, the Mann–Whitney non-parametric test was applied. To compare more than 2 data sets with Gaussian distribution, the parametric ANOVA (One-way Analysis of Variance) test was applied, and the Bonferroni test was applied for multiple comparisons. To compare more than 2 data sets with non-Gaussian distribution, the Kruskal–Wallis test, a non-parametric test, was applied, and for multiple comparisons, Dunn’s test was applied. The Chi-square test was applied to determine the association between qualitative variables. We applied univariable and multivariable logistic regression for dependent variables with binary classification (Yes or No) and univariable and multivariable ordinal logistic regression for dependent variables with ordinal classification. The Pearson test was applied to evaluate the correlation (to measure the strength of association) between quantitative variables. To estimate the survival, we used Kaplan–Meier curves. The significance threshold chosen for the *p*-value was 0.05. The statistical analysis was performed using SPSS software version 29.0 (SPSS, Chicago, IL, USA).

## 3. Results

### 3.1. Patients Characteristics

This study included 128 patients who were divided into four groups: 

(1) patients with both sarcopenia and frailty;

(2) patients without sarcopenia and with frailty;

(3) patients with sarcopenia and without frailty;

(4) patients without sarcopenia and frailty.

The mean age for patients without sarcopenia and frailty was higher (62.70 ± 9.673 years) compared to those with sarcopenia and frailty (55.76 ± 10.46 years) (Table 1). We did not obtain a significant statistical value comparing the mean age for these four groups, including in the Bonferroni test.

The majority of the male patients in this study were without sarcopenia and frailty (63.93%), compared to those with both conditions. Similarly, most females had no signs of sarcopenia and frailty (36.07%) (Table 1). The Child–Pugh score was calculated for all patients, revealing that those with both sarcopenia and frailty most frequently had a C score (69.70%). Meanwhile, those who had sarcopenia without frailty predominantly had a B score (45.83%). Patients without sarcopenia or frailty had a higher prevalence of A scores, at 63.93%. This distribution suggests a correlation between the presence of these conditions and altered Child–Pugh scores. Additionally, MELD-Na scores were higher in patients with both sarcopenia and frailty, with an average of 25.45 ± 6.924, compared to 14.95 ± 5.640 in those without these conditions (Table 1). Notably, patients with both conditions had higher values of MELD-Na scores of 21.84 ± 6.951, significantly higher than the 14.95 ± 5.640 in those without sarcopenia or frailty (*p* < 0.0001). These findings highlight a significant relationship between frailty, sarcopenia, and the associated scores.

For both scores, we obtained significant statistic values (*p* < 0.0001) (Table 1). BMI values did not significantly influence statistics in the four groups of patients. For all four groups of patients, HGS, SMAI, LFI, and SPPB were calculated. In the group of patients with sarcopenia and frailty and the group with sarcopenia and without frailty, values of HGS were lower (19 kg/m^2^) compared to the group without sarcopenia and with frailty and the group without both sarcopenia and frailty (28 kg/m^2^) (*p* < 0.0001) (Table 1).

The mean value of SMI was higher in the group without sarcopenia and frailty (49.77 ± 5.836 m^2^), while the lowest values were recorded in the group with sarcopenia and frailty (40.20 ± 4.836 m^2^) (Table 1). Significative statistical values were obtained in this case also (*p* < 0.0001). The LFI score also showed significant differences, with higher mean values in the group with sarcopenia and frailty (4.897 ± 0.267) and the lowest in the group without sarcopenia and frailty (3.651 ± 0.368). SPPB was applied to all four groups, and we recorded high values in the group without sarcopenia and frailty (10.85 ± 1.621) (Table 1). Moreover, in our study, higher MELD-Na scores were correlated with lower HGS values (−0.2354, 95% CI: −0.3929 to −0.06444, *p* = 0.0075) and higher LFI values (0.6270, 95% CI: 0.5088 to 0.7220, *p* < 0.0001) (Table 2).

Biomarkers like albumin, sodium, C-reactive protein, bilirubin, and platelets were also considered. Albumin, sodium, and platelet levels were significantly lower in the group with both sarcopenia and frailty than in those without these two complications. Values of CRP and bilirubin were higher in the group with both sarcopenia and frailty vs. those without both sarcopenia and frailty. Encephalopathy was present in a higher percentage in the group with sarcopenia and frailty (51.52%). Infections were recorded in all four groups, and spontaneous bacterial peritonitis obtained significant statistical values (*p* = 0.0389) (Table 1). Readmission in the hospital was recorded more often in the group with sarcopenia and frailty (66.67%) compared to the other three groups (*p* < 0.0001) (Table 1). In-hospital death was higher in the group with sarcopenia and frailty (27.27%), and this trend was maintained at 6 and 12 months (Table 1).

**Figure 1:**
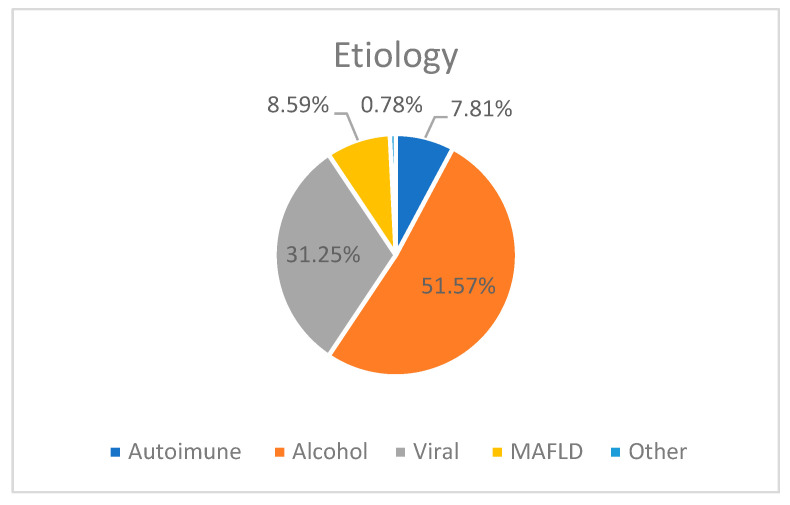
The etiology of chronic liver disease in our study groups.

In all four groups, the most frequent etiology of chronic liver disease was alcohol (51.56%), followed by viral (31.25%) and MAFLD (8.59%) (Figure 1).

In our study group, the prevalence of sarcopenia was 44.53% (57 patients) and frailty was 32.81% (42 patients). Both were recorded in 25.78% of cases (33 patients).

### 3.2. Short- and Long-Term Survival of Patients with Sarcopenia and Frailty

**Figure 2:**
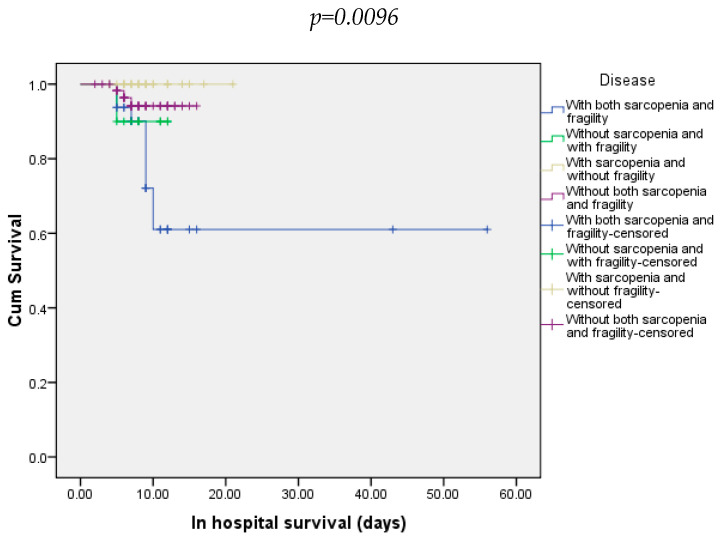
In-hospital survival of patients included in all four groups.

During hospitalization, 10.2% of all patients died. Regarding all groups of patients, death occurred in 27.3% of those with both sarcopenia and frailty, 10% in those without sarcopenia and with frailty, and 4.92% in those without sarcopenia and/or frailty (Figure 2).

**Figure 3:**
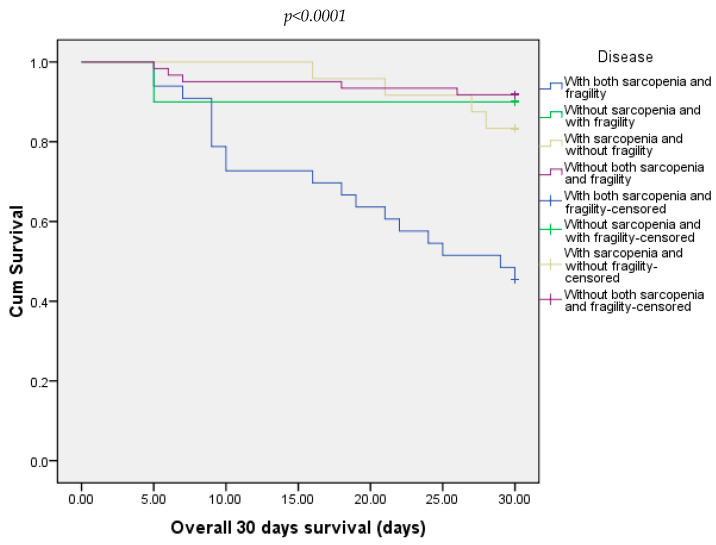
Overall survival of patients included in all four groups at 30 days.

**Figure 4:**
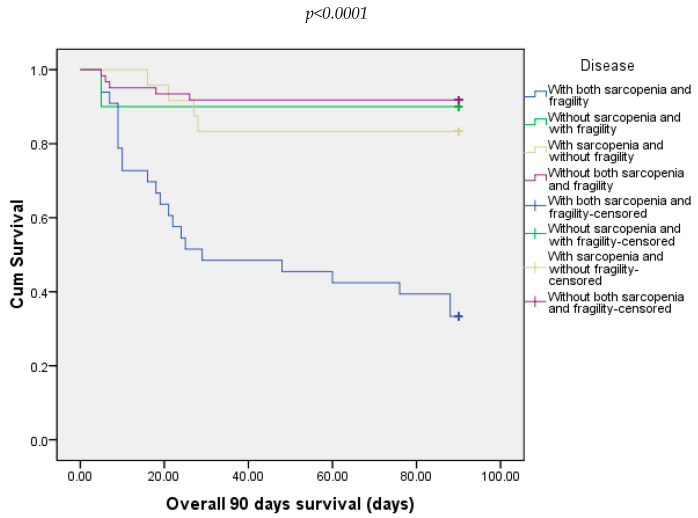
Overall survival of patients included in all four groups at 90 days.

At 30 days, 21.88% of all patients died. Most of them were from the group with both sarcopenia and frailty (54.54%) and 8.20% from the group without both sarcopenia and frailty (Figure 3). At 90 days, 25% of all patients died. In this case, the majority were from the group with sarcopenia and frailty (66.66%). The other three groups did not record any deaths (Figure 4).

**Figure 5:**
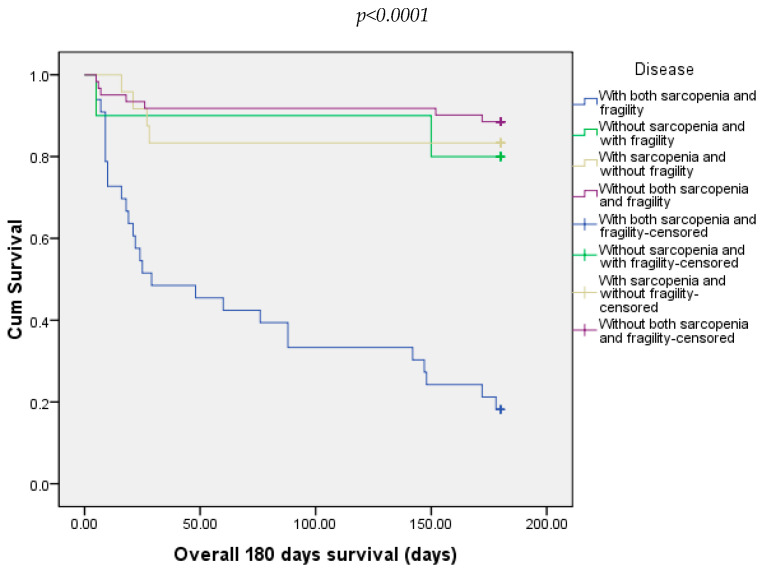
Overall survival of patients included in all four groups at 180 days.

In our study, at 180 days, we recorded 31.25% of deaths. Most of the deaths were in the groups with both sarcopenia and frailty (81.81%), followed by 11.48% from the group without both sarcopenia and frailty and 16.67% from those with sarcopenia and without frailty (Figure 5).

**Figure 6:**
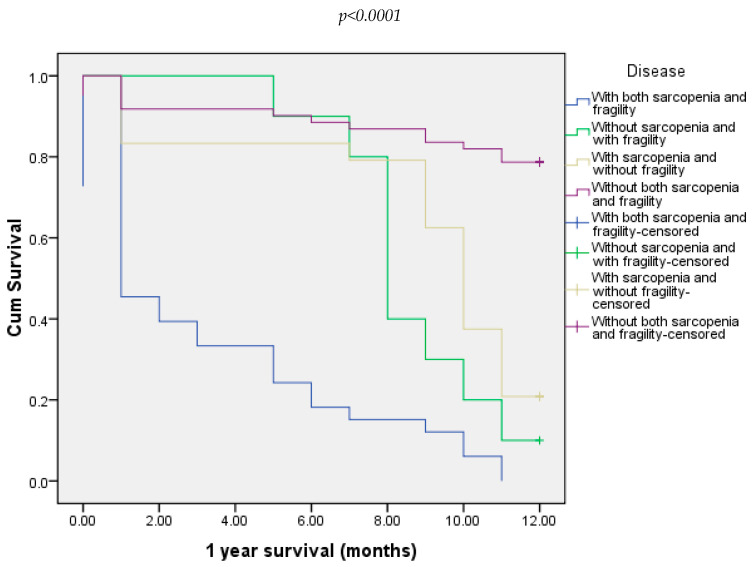
Overall survival of all four groups of patients at 12 months.

Twelve-months follow-up showed that death occurred in 57.81% of all patients. Regarding every group, all patients with both sarcopenia and frailty died. Also, death occurred in 90% of the patients from the group without sarcopenia and with frailty and in 78.2% of the patients in the group with sarcopenia and without frailty (Figure 6).

We compared all four groups, and the univariable analysis revealed that increased values of MELD-Na, Child–Pugh score, bilirubin, and the presence of PBS are associated with poor outcomes (Table 3). In addition, low levels of albumin, sodium, platelets, and high values of CRP in all four groups were associated with high mortality. Multivariable analysis showed that the MELD-Na value is an independent factor for overall survival (Table 3). According to univariable ordinal regression, etiology was not a prognostic factor for patient survival in all four groups (Table 4).

## 4. Discussion

Our study included 128 patients diagnosed with advanced liver disease and with or without the presence of sarcopenia or frailty. All patients were admitted as an emergency due to decompensated liver disease or associated infections.

We divided the patients into four groups based on their presence of frailty or sarcopenia and analyzed different parameters that could influence their outcomes.

The assessment of these two complications is essential not just for liver transplantation but also to increase the quality of life and survival of these patients [5,11,18,20]. The assessment is not complicated, but extra time is required for the clinician to take all the anthropometric measurements to establish the diagnosis [22].

Data in the literature are limited because very few studies have assessed both sarcopenia and frailty [16,18,19,20]. Another inconvenience is that no studies use all the parameters to define frailty and sarcopenia. Both are interrelated and are usually present in the same patient [18,19].

In our study group, diagnosis of frailty was defined as the presence of three of the five criteria, unlike other studies, which established the diagnosis just by LFI [18]. For the diagnosis of frailty, we used HGS and LFI. The prevalence of frailty was 32.8%, double that reported by other studies [18,26]. The literature showed that frailty is common and affects 17–43% of patients with advanced liver disease [16,18,26]. Frailty has an essential role in predicting morbidity and mortality in patients with cirrhosis. More than that, it can predict hospitalization and the length of hospitalization [16,18,20,26].

The prevalence of sarcopenia in our group was 44.53%, which is similar to that reported in the EASL guidelines [23]. Although the skeletal muscle index (SMI) assessed by CT scan is the gold standard to identify sarcopenia among patients with cirrhosis, we made the diagnosis based on decreased HGS, decreased physical performance (SPPB), and measurement of SMI, unlike other studies [6,16,18,19,20]. The cut-off thresholds for SMI were <50 cm^2^/m^2^ for men and <39 cm^2^/m^2^ for women [27,28]. Handgrip strength is useful in assessing the contractile function of skeletal muscles. It is a simple, cheap, and effective method to detect malnutrition in patients with cirrhosis [6,18,19,20,27,28,29]. At the same time, it can predict the incidence of significant complications and mortality in the short and long term [10,23,27,28,30,31].

Sarcopenia often coexists with frailty, and in our study we identified that 25.78% of patients had both. Our findings are consistent with those of other studies, although they typically focused on patients listed for liver transplantation [10,11,18]. It is important to note that the absence of sarcopenia does not rule out frailty. In our study, 7.81% of frail patients were without sarcopenia. Therefore, it is crucial to screen for both pathologies, as their underlying mechanisms differ [18,19,20].

We also observed that younger patients were more likely to present with both sarcopenia and frailty (55.76 ± 10.46 years) compared to those without both sarcopenia and frailty (62.70 ± 9.673 years). Additionally, alcohol was the most common etiology among our patient groups, accounting for 51.56%. Alcohol consumption is frequently associated with malnutrition [31], and advanced liver disease due to alcohol is more often associated with an increased prevalence of both frailty and sarcopenia.

It is well-known that high-value scores of Child–Pugh and/or MELD-Na scores are more frequently associated with both sarcopenia and frailty [16,18,19]. Our study confirmed these data, showing that high scores of Child–Pugh and MELD-Na were more often associated with groups with both sarcopenia and frailty than those without both sarcopenia and frailty (*p* < 0.0001).

In addition, patients with both sarcopenia and frailty are associated with lower albumin levels, which are attributable not only to cirrhosis but also to malnutrition (*p* = 0.0060). Interestingly, frail patients without sarcopenia had lower albumin values (3.080 ± 0.547) compared to those with sarcopenia and without frailty (3.294 ± 0.398). This suggests that frailty is more strongly associated with lower albumin values.

Our study also found that patients with both sarcopenia and frailty had more frequent low sodium and platelet values, as well as elevated bilirubin levels compared to those without sarcopenia and/or frailty.

Patients with both frailty and sarcopenia were more frequently associated with hepatic encephalopathy and spontaneous bacterial peritonitis compared to those without these two complications.

Elevated CRP values, commonly associated with infection, were also linked to worse outcomes in cirrhotic patients with both sarcopenia and frailty compared to those without both of these complications (*p* = 0.0039).

Furthermore, patients with both sarcopenia and frailty had higher rates of readmission within 30 days of discharge, as well as increased mortality at 30 days, 90 days, 180 days, and 12 months. This suggests that increased systemic inflammation contributes to higher mortality rates both during hospitalization and in the short and long term. In all four patient groups, higher values of MELD-Na, Child–Pugh score, bilirubin, and the presence of PBS were associated with poor outcomes. Lower albumin, sodium, and platelet levels, in addition to high values of CRP, were also linked to higher mortality in all groups, with the greatest impact in the group with both sarcopenia and frailty. One study that focused on patients with both sarcopenia and frailty also showed that low values of albumin and sodium were associated with higher mortality in these patients [18]. Additionally, they found that creatinine levels and neutrophiles/lymphocytes ratio influenced outcomes in this group of patients [18]. The key difference in their study was that they considered only the Child–Pugh score and MELD rather than MELD-Na

In our study, the mortality rate was 2.54 times higher in the group with both sarcopenia and frailty than in those without both of them. Additionally, overall survival was 2.11 times higher in the group without sarcopenia and with frailty than in those with sarcopenia and without frailty. These findings highlight the synergistic role of these two complications in the unfavorable evolution of cirrhotic patients, as observed by Guo et al. in their study [18].

However, our study has both strengths and limitations.

A strength is that we included at least three types of measurements to establish the diagnosis of sarcopenia and frailty. The use of HGS and SMI measurements via CT scans enhances the accuracy of the diagnosis.

One limitation is that this study was conducted in a single center with a relatively small patient sample. Additionally, we only included patients capable of performing exercise, excluding those with severe decompensated liver disease. This exclusion may have introduced selection bias, as this patient subgroup is significant in advanced liver disease. Future research should address this limitation to provide a more comprehensive understanding. Another limitation is literature-based cut-off values, which may not apply to different populations.

We propose expanding this study to include a larger cohort with a longer follow-up period, integrating targeted therapeutic interventions to improve sarcopenia and nutritional status.

## 5. Conclusions

The presence of sarcopenia and frailty significantly impacts patients with advanced liver disease, contributing to increased mortality rates. Early identification of these conditions allows timely intervention to improve short- and long-term outcomes. Nutritional support, exercise, and cognitive interventions are crucial in addressing these complications.

## Figures and Tables

**Table 1:** The general characteristics of patients included in this study were divided into four groups according to the presence/absence of sarcopenia and frailty.

		With Sarcopenia + Frailty (*n* = 33)	Without Sarcopenia + with Frailty (*n* = 10)	Sarcopenia + Without Frailty (*n* = 24)	Without Sarcopenia + Frailty (*n* = 61)	*p*-Value
Age		55.76 ± 10.46 (56.00)	62.70 ± 9.673 (64.00)	57.79 ± 11.54 (58.50)	57.33 ± 12.83 (57.00)	0.4464
Gender	Female	12 (36.36%)	4 (40.00%)	9 (37.50%)	22 (36.07%)	0.9956
	Male	21 (63.64%)	6 (60.00%)	15 (62.50%)	39 (63.93%)	
Child–Pugh score	A	3 (9.09%)	1 (10.00%)	7 (29.17%)	29 (47.54%)	<0.0001
	B	7 (21.21%)	3 (30.00%)	11 (45.83%)	20 (32.79%)	
	C	23 (69.70%)	6 (60.00%)	6 (25.00%)	12 (19.67%)	
Child–Pugh scores values		10.58 ± 2.550 (10.00)	9.500 ± 2.014 (10.00)	8.083 ± 1.692 (8.000)	7.492 ± 1.988 (7.000)	<0.0001
MELD-Na		25.45 ± 6.924 (27.00)	20.40 ± 5.680 (22.00)	17.46 ± 4.433 (17.00)	14.95 ± 5.640 (14.00)	<0.0001
BMI		25.01 ± 3.803 (24.92)	23.57 ± 3.141 (23.83)	25.17 ± 3.080 (25.19)	25.28 ± 2.801 (25.18)	0.4696
HGS		16.73 ± 4.869 (19.00)	24.40 ± 7.961 (28.00)	18.25 ± 5.194 (19.00)	27.28 ± 5.905 (28.00)	<0.0001
SMI		40.20 ± 4.836 (41.98)	49.04 ± 7.113 (52.19)	42.15 ± 5.889 (43.99)	49.77 ± 5.836 (51.26)	<0.0001
LFI		4.897 ± 0.267 (4.900)	4.575 ± 0.099 (4.540)	4.172 ± 0.219 (4.185)	3.651 ± 0.368 (3.690)	<0.0001
SPPB		5.636 ± 1.496 (5.000)	6.300 ± 1.767 (6.000)	9.083 ± 1.349 (9.000)	10.85 ± 1.621 (12.00)	<0.0001
Albumin		2.877 ± 0.480 (2.900)	3.080 ± 0.547 (2.985)	3.294 ± 0.398 (3.320)	3.222 ± 0.542 (3.260)	0.0060
Natrium		130.2 ± 5.758 (129.0)	132.3 ± 3.945 (132.0)	135.5 ± 4.061 (135.5)	135.2 ± 3.459 (136.0)	<0.0001
CRP		13.46 ± 14.47 (9.800)	10.65 ± 10.53 (7.750)	7.401 ± 6.614 (6.240)	6.788 ± 8.512 (4.200)	0.0039
Platelets		78,848 ± 36,679 (67,000)	90,400 ± 28,508 (97,000)	110,000 ± 55,318 (102,500)	115,623 ± 53,087 (102,000)	0.0019
Total bilirubin		6.598 ± 6.466 (4.510)	4.268 ± 4.179 (2.750)	3.123 ± 2.601 (2.800)	2.769 ± 3.129 (1.700)	<0.0001
Encephalopathy	Present	17 (51.52%)	6 (60.00%)	5 (20.83%)	12 (19.67%)	0.0017
	Absent	16 (48.48%)	4 (40.00%)	19 (79.17%)	49 (80.33%)	
Refractor ascites	Present	5 (15.15%)	1 (10.00%)	4 (16.67%)	8 (13.11%)	0.9506
	Absent	28 (84.85%)	9 (90.00%)	20 (83.33%)	53 (86.89%)	
Pneumonia	Present	8 (24.24%)	1 (10.00%)	2 (8.33%)	7 (11.48%)	0.2662
	Absent	25 (75.76%)	9 (90.00%)	22 (91.67%)	54 (88.52%)	
UTI	Present	11 (33.33%)	3 (30.00%)	5 (20.83%)	11 (18.03%)	0.3728
	Absent	22 (66.67%)	7 (70.00%)	19 (79.17%)	50 (81.97%)	
SBP	Present	11 (33.33%)	3 (30.00%)	5 (20.83%)	6 (9.84%)	0.0389
	Absent	22 (66.67%)	7 (70.00%)	19 (79.17%)	55 (90.16%)	
Hospitalization days		11.36 ± 10.38 (9.000)	8.500 ± 2.799 (8.000)	9.417 ± 4.180 (8.500)	8.459 ± 3.134 (8.000)	0.6389
Re-admissions		22 (66.67%)	3 (30.00%)	18 (75.00%)	5 (8.20%)	<0.0001
30-days readmissions		16.82 ± 9.106 (16.50)	22.67 ± 2.517 (23.00)	23.44 ± 7.180 (26.00)	24.20 ± 8.167 (27.00)	0.0582
In-hospital deaths		9 (27.27%)	1 (10.00%)	0 (0.00%)	3 (4.92%)	0.0017
180-day deaths		27 (81.82%)	2 (20.00%)	4 (16.67%)	7 (11.48%)	<0.0001
12-month deaths		33 (100.00%)	9 (90.00%)	19 (79.17%)	13 (21.31%)	<0.0001

MELD-Na—the Model for End-Stage Liver Disease–Natrium; BMI—body mass index; HGS—handgrip strength; SMI—skeletal muscle index; LFI—liver frailty index; SPPB—Short Physical Performance Battery Protocol; CRP—C-reactive protein; UTI—Urinary Tract Infection; SBP—spontaneous bacterial peritonitis.

**Table 2:** The Pearson correlation of MELD-Na with HGS and LFI.

	MELD-Na		
	Pearson r	95% Confidence Interval	*p*-Value
HGS	−0.2354	−0.3929 to −0.06444	0.0075
LFI	0.6270	0.5088 to 0.7220	<0.0001

MELD-Na—the Model for End-Stage Liver Disease–Natrium; HGS—handgrip strength; LFI—liver frailty index;.

**Table 3:** The results of the univariable and multivariable analyses for independent survival factors in all four groups of patients included in this study.

All Four Groups	Univariable Analysis			Multivariable Analysis		
	Estimate	95% Confidence Interval	*p*-Value	Estimate	95% Confidence Interval	*p*-Value
Age	−0.003	−0.030 to 0.024	0.8314	0.020	−0.013 to 0.053	0.2253
Gender	−0.033	−0.700 to 0.635	0.9238			
MELD-Na	0.208	0.146 to 0.270	<0.0001	0.192	0.087 to 0.297	0.0003
Child–Pugh	0.480	0.316 to 0.644	<0.0001	0.119	−0.224 to 0.462	0.4961
Albumin	−0.950	−1.600 to −0.301	0.0041	0.255	−0.686 to 1.195	0.5957
Na	−0.185	−0.262 to −0.107	<0.0001			
CRP	0.055	0.017 to 0.094	0.0051	0.003	−0.041 to 0.047	0.9024
Platelets	−1.203 × 10^3^	−1.940 × 10^3^ to −4.668 × 10^3^	0.0014			
Bilirubin	0.168	0.076 to 0.260	0.0004			
Encephalopathy	1.226	0.517 to 1.934	0.0007			
Refractory ascites	0.109	−0.813 to 1.031	0.8175			
Pneumonia	0.710	−0.207 to 1.627	0.1290	−0.369	−1.732 to 0.994	0.5959
UTI	0.653	−0.102 to 1.408	0.0900	0.0004	−1.302 to 1.303	0.9994
SBP	1.171	0.350 to 1.992	0.0052	0.269	−0.656 to 1.957	0.5687

MELD-Na—the Model for End-Stage Liver Disease-Natrium; Na—Natrium; CRP—C-reactive protein; UTI—Urinary Tract Infection; SBP-spontaneous bacterial peritonitis.

**Table 4:** The univariable analysis outcomes for survival factors across all four patient groups, stratified by etiology.

Four Groups	Univariable Analysis		
	Estimate	95% Confidence Interval	*p*-Value
Alcohol	−0.145	−0.790 to 0.500	0.6586
Viral	0.294	−0.398 to 0.986	0.4047
Metabolic	0.532	−0.600 to 1.664	0.3572
Autoimmune	−0.872	−2.146 to 0.401	0.1794

## Data Availability

The original contributions presented in this study are included in the article. Further inquiries can be directed to the corresponding authors.
